# The resin bee subgenus Ranthidiellum in Thailand (Megachilidae, Anthidiini): nesting biology, cleptoparasitism by *Stelis*, and new species

**DOI:** 10.3897/zookeys.1031.57836

**Published:** 2021-04-15

**Authors:** Pakorn Nalinrachatakan, Prapun Traiyasut, Anupong Khongnak, Manop Muangkam, John S. Ascher, Natapot Warrit

**Affiliations:** 1 Chulalongkorn University, Bangkok, Thailand Chulalongkorn University Bangkok Thailand; 2 Ubon Ratchathani Rajabhat University, Ubon Ratchathani, Thailand Ubon Ratchathani Rajabhat University Ubon Ratchathani Thailand; 3 National University of Singapore, Singapore, Singapore National University of Singapore Singapore Singapore

**Keywords:** *
Anthidiellum
*, *
Malanthidium
*, pollinator, systematics, taxonomy, wool carder bee

## Abstract

Resin bees of the subgenus Ranthidiellum, are rare and endemic to Southeast Asia. These bees are known to construct resinous entrance tubes to their nests. Here, the new species Anthidiellum (R.) phuchongensis**sp. nov.** is described along with a description of its nest collected from Phu Chong Na Yoy National Park, Ubon Ratchathani Province, Thailand. In addition, the bee cleptoparasite, Stelis (Malanthidium) flavofuscinular**sp. nov.**, and the male of A. (R.) ignotum Engel, 2009, are described for the first time. A key to *Ranthidiellum* species is also provided.

## Introduction

Bees in genus Anthidiellum Cockerell, 1904 subgenus Ranthidiellum Pasteels, 1969 are rare, enigmatic, and restricted to Southeast Asia with only four reported species (Engel 2009; Ascher and Pickering 2020): A. (R.) apicepilosum (Dover, 1929), A. (R.) meliponiforme (Cockerell, 1919), A. (R.) rufomaculatum (Cameron, 1902), and the most recent A. (R.) ignotum Engel, 2009. *Ranthidiellum* bees are robust, of a moderate size with a reddish or fulvous infused integument, and possess an arcuate subantennal suture. Females of *Ranthidiellum* are equipped with an apically broad mandible (> 1.5× the base width), whereas an apical comb on S5 and median spine on T7 can be found in males (Pasteels 1969, 1972; Michener 2007). *Ranthidiellum* morphology was hypothesized to mimic its sympatric stingless bees (Cockerell 1919; Soh et al. 2016) and is clearly distinct from its most common sympatric congeners, such as A. (Pycnanthidium) smithii (Ritsema, 1874), which is a small bee with an overall black body and yellow maculation. Engel (2009) recently provided a provisional key to female *Ranthidiellum* species.

As *Ranthidiellum* species are rarely collected, they were not included in the recent phylogenetic studies of the Tribe Anthidiini (Combey et al. 2010; Gonzalez et al. 2012; Litman et al. 2016). Thus, insight into their evolution, together with their morphology, life history, and behavior are poorly understood. Pagden (1934) discovered the only known nests of A. (R.) apicepilosum in Bukit Kutu, Malaysia, which were burrowed in abandoned mud wasp nests using resins. Pasteels (1972) later provided supplementary details for this finding.

During a field collecting trip in October 2018 at Phu Chong Na Yoy National Park (PCNYNP), Ubon Ratchathani Province, Thailand, we discovered a small congregation of *Ranthidiellum* nests on a vertical earth bank. The nests were then excavated and brought back to the laboratory at the Ubon Ratchathani Rajabhat University, and reared until adult bees emerged. Here, we describe a new *Ranthidiellum* species discovered at PCNYNP, and also describe the cleptoparasitic bees that emerged from the host cells in the nest. Since the new *Ranthidiellum* described is morphologically similar to A. (R.) ignotum Engel, 2009, we examined additional *Ranthidiellum* material to facilitate comparison between the two, and a description of the male A. (R.) ignotum for the first time.

## Material and methods

**Figure 1.**
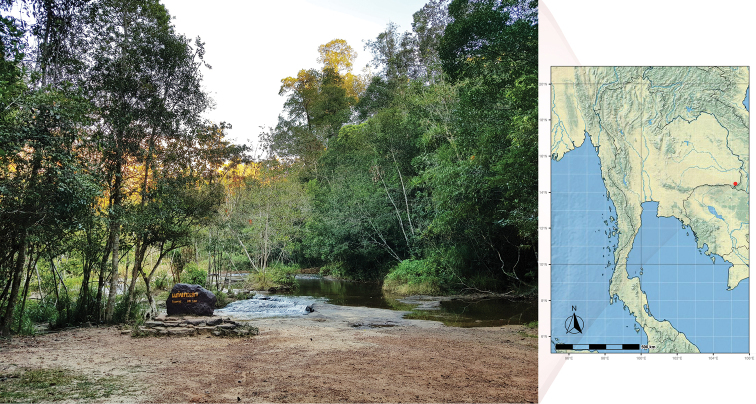
Study site at Kaeng Ka Lao Stream, PCNYNP, Ubon Ratchathani Province (Thailand), with the map produced using QGIS (3.16.0).

*Ranthidiellum* nests were discovered on a sandy earth bank on a walking trail leading to Kaeng Ka Lao Stream (Figs 1 and 8) [14°26'10.98"N, 105°16'37.05"E, alt. 322 m], PCNYNP, Ubon Ratchathani Province in October 2018 (we revisited the site again in December 2018 and 2019). Adjacent the Kaeng Ka Lao Stream is a secondary dipterocarp forest. All eight active nests (seven from 2018 and one from 2019) were carefully excavated from the earth bank using brushes and small hand shovels. A couple of abandoned nests were also found in the adjacent area. The collected nests were wrapped in cotton wool, clumped paper, and saran (‘cling’) wrap before being put in a field box and transported back to the laboratory for examination.

Dissection of the seven nests collected from 2018 revealed a total of 18 individuals (six larvae, nine pupae, and three quiescent adults). Eight adult bees were collected: 2♀ from outside of the nesting area and 6 (3♀, 3♂) from the reared nest (see below), and were deposited at the Chulalongkorn University Natural History Museum (**CUNHM**) for morphological examination. Seven additional specimens of Anthidiellum (Ranthidiellum) ignotum Engel, 2009 (6♀, 1♂) deposited at **CUNHM** and the Department of Entomology and Plant Pathology, Chiang Mai University, Thailand (**CMU**), were also examined and compared with the collected individuals.

A single nest from 2019 was maintained in a plastic box under room temperature and relative humidity (22–25 °C, 70–90%) at Ubon Ratchathani Rajabhat University. Water spray was used to keep the humidity inside the box relatively constant. The first bee emerged after day 47. The time of eclosion of each remaining bee was recorded.

*Ranthidiellum* specimens were examined under light microscopy (Zeiss Stemi 508 stereomicroscope). Photographs were taken using a Canon 7D Mark II digital camera control via Canon EOS Utility software, attached to the stereomicroscope. All photographs were post-processed using Adobe Photoshop CC 2018 and Adobe Lightroom CC 2018 software. All terminology and abbreviations used follow Engel (2009), Kasparek (2015), Michener (2007), Michener and Griswold (1994), and Michener et al. (1994). Male specimens were dissected to reveal the genitalic structures using a protocol modified from Gonzalez et al. (2012). To clear most of the artifacts, we altered the process by immersing the genitalia in 3M KOH at room temperature (24 °C) for 20 h, or heating in hot water until ready to be dissected.

Photos or images of type specimens of A. (R.) apicepilosum Dover, 1929 (NHML 014026685), A. (R.) meliponiforme (Cockerell, 1919) (NHML 014026114), and A. (R.) rufomaculatum (Cameron, 1902) (NHML 014026141) at the Natural History Museum, London, UK (**NHMUK**), were examined through the “Apoidea (Bee) Type Digitization Project” digital platform from https://data.nhm.ac.uk/, and the images are provided by Mr. Chawatat Thanoosing and Ms. Natalie Dale-Skey (**NHMUK**).

## Systematics

### Genus *Anthidiellum* Cockerell, 1904

#### Subgenus Ranthidiellum

Taxon classificationAnimaliaHymenopteraMegachilidae

Pasteels, 1969

1A86F2B0-FED8-544D-BEF0-A9161748AC5A

Anthidiellum (Ranthidiellum) Pasteels, 1969: 48. Type species: Protoanthidium rufomaculatum Cameron, 1902, by original designation. [other aspects of type designation discussed in Michener and Griswold (1994)]
Anthidiellum (Rhanthidiellum) Pasteels, 1972: 102, unjustified emendation of Ranthidiellum Pasteels, 1969.

##### Diagnosis.

Moderate size (around 7–10 mm); clypeus subtriangular as frontoclypeal suture curved upwards; subantennal suture arcuate; eyes convergent ventrally; preoccipital margin round, not carinate; pronotal lobe raised, extended, and lamellated; omaular carina complete to the ventral region of thorax; scutellum and axilla large, margin translucent; propodeum without dimple; abdomen appears oval, shiny with reddish, orangish, or ferruginous extended.

***Female*:** mandible apically broad, about 1.5× wider than base, with four small teeth; hind basitarsus enlarged; abdomen oval shaped, longer than wide, gradually smaller from third segment; T6 margin subtruncate, shield-like; S6 simple.

***Male*:** mandible tridentate, apex not wide as in female; T6 with apical transverse border; T7 short with median spine; S4 marginally with transparent membrane; S5 indented with black comb; gonoforceps bifid.

#### Anthidiellum (Ranthidiellum) ignotum

Taxon classificationAnimaliaHymenopteraMegachilidae

Engel, 2009

79ED3088-B233-51B4-9D8B-D817B752BB49

**Figure 2.**
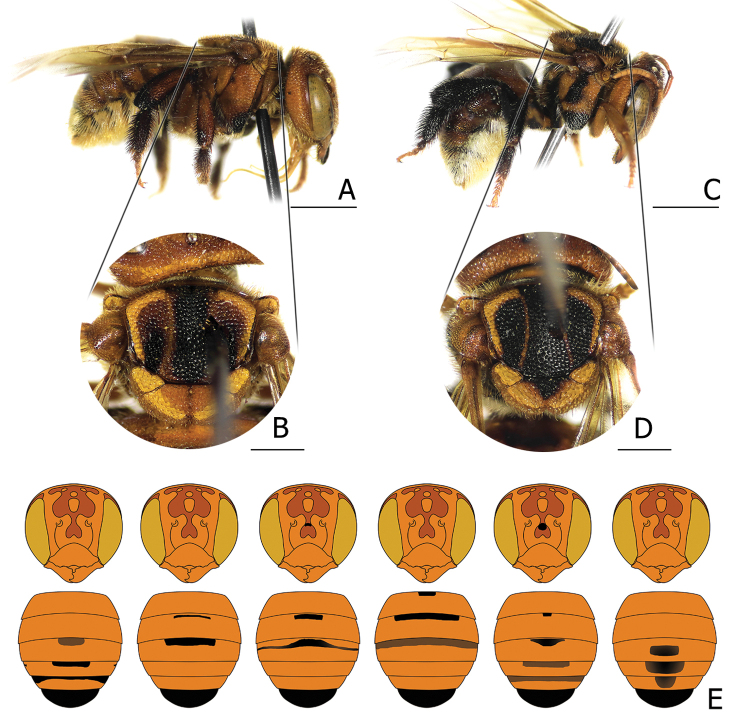
Female *Anthidiellum ignotum* Engel, 2009 **A, B** lateral habitus and mesosoma of the “less melanized” individual (BSRU-AA-1250) **C, D** lateral habitus and mesosoma of an individual with a black scutellum mark (BSRU-AA-6709) **E** female faces and metasoma, showing variations of black infused stigma. From left to right: BSRU-AA-2668, 1250, 1249, 6708, 6709, and CMU-0013. Scale bars: 2 mm (**A, B**) or 1 mm (**C, D**).

Figs 2 , 4 (right), 5 (right)

Anthidiellum ignotum Engel, 2009: 30–34. (♀, holotype)
Anthidiellum ignotum Engel: Soh et al. 2016, 55. (♀)

##### Material examined

7 (6♀, 1♂). Thailand: Chiang Mai (new record), 2♀, 1♂, Chiang Dao, Pha Dang National Park, Srisuwan Waterfall, Suan Dok Mai (19°37'49.88"N, 98°57'12.40"E, alt. 527.96 m), 19 Dec. 2018, N. Warrit et al. (CUNHM: BSRU-AA-6708, 6709). 1♀, Mae Chaem District, Highway 1088, 9 Dec. 2016, N. Warrit et al. (CUNHM: BSRU-AA-2668). 1♀, Samoeng, 13 Dec. 1992, Wichai [initially identified as “Apidae” by Wichit] (CMU-0013); 2♀, Phayao, Mueang, Maeka, Phayao University, 1 Jun. 2012, W. Suwannarak et al. [CUNHM: BSRU-AA-1249, 1250, same specimens in Soh et al. (2016)].

##### Distribution.

Thailand [Chiang Mai (Chiang Dao, Mae Chaem, and Samoeng Districts) and Phayao (Maeka District) Provinces; Nakhon Ratchasima Province, Sakaerat Environment Research Area (type locality from Engel (2009))].

##### Diagnosis.

This species can be distinguished from other *Ranthidiellum* by its remarkably bright ferruginous color, mostly without a black integument on their faces; tergites red-brown on marginal zone; T5 and T6 covered with pale-golden short tomentum; leg with reddish integument on coxa and femur of midleg and hindleg (see Engel 2009). Male with more extensive black maculation, especially on scutum and metasoma, whereas overall brighter than in female, predominantly on scutum; S4 gradulus incomplete (Fig. 5D); gonoforceps bifid, with extended medio-lateral carina with acute sharp edge.

##### Description.

♂: ***Structure***: Length 7.8 mm, head width 3.2 mm, intertegulae distance 2.2 mm, wingspan 15.2 mm.

***Head*.** Overall prosomal coloration somewhat lighter than in female. Head lightly orange to yellow, gradually increasing in brightness ventrally, sparsely covered with bright yellow hairs. Maculation as in female but with dark contrast (Fig. 4H): inverted heart-shaped mark on paraocular area making median Y-shape bridge connecting two large ovoid marks above antennal socket, extending upwards, concatenated at ocellar triangle, and forming a transverse band on vertex. Eye margin with narrow black mark on dorsal margin to about half of outer orbital margin. Clypeus bright yellow. Mandible yellow, shiny, apex not as broad as in females. Outer ridge conspicuous making upper area shallowly depressed. Teeth black, tridentate with large acute tooth at apex. Labrum dark yellowish, without conspicuous large hairs on surface as in female. Scape orange to yellow. Pedicel brown. Antennal flagellum orange brown on 1^st^ and basal half of 2^nd^ segments, other flagellomere pale light brown with pits on front and shiny yellow surface without pits posteriorly.

***Mesosoma*.** Scutum largely black, with yellow inverted L-shaped band on anterolateral margin extending to fine paramedial line and abutting posterior margin, slightly curved medially. Scutellum and axilla yellow, median with black inverted triangular shape. Tegula dark yellow, somewhat translucent. Pronotal lobe pale yellow, strongly carinate to lamellate. Metanotum yellowish, laterally black. Propodeum black with small orange spot anteriorly around propodeal spiracle. Anterior surface of mesepisternum black, lateral surface yellow-orange with extensive black marks dorsally and in area adhering to metepisternum. Metepisternum yellow-orange, black on anterior and dorsal margin. Overall mesosoma covered with bright yellow hairs, except for pale white hairs on lower part of mesepisternum.

***Wings*.** Forewing basally infuscated as in female, but with obvious hyaline patch covering parts of radial cell, 1^st^ medial cell, and parts of 1^st^ submarginal cell. Also, largely subhyaline on the marginal, submarginal, and 2^nd^ medial cells.

***Legs*.** Foreleg yellow-orange, with black mark on upper part of coxa, and small basal mark on both anterior and posterior surfaces of trochanter and femur. Midleg and hindleg largely black with some obscure red-brown infused, except for dark yellow on middle coxa, posterior area on hind coxa dark brown, apical area of middle trochanter, especially on posterior surface, yellow-brown, upper and lower parts of middle femur with obscure yellow-brown band, and middle tibia with outer brown-yellowish band. Middle and hind tarsi dark brown to black but gradually lighter towards end. Claw dark yellowish to brown, black on both apical and subapical tooth. Arolia present, hair bright yellow on foreleg, the rest overall black but white on dorsal part of coxa, femur, and trochanter of midleg and hindleg. Tarsal hair generally dark brown, gradually becoming bright yellow at the end.

***Metasoma*.** Yellow-orange with thin black band infused at basal terga. Apical margin subhyaline showing black area of the former. T1 black on frontal surface defined with carina, extended to upper lateral surface. T2–T5 with small lateral dots and black thin stripes on basal part. T6 apically curved inward, forming conspicuous apical border with black surfaces on back (Fig. 4F). T7 shield-like, black at margin, with median acute apex. Dorsal surfaces shiny and glabrous. Sternites overall yellow-orange except dark brown on S1. S4 (Fig. 5D) margin extended as transparent membrane, median of margin with two small black teeth. S5 widely emarginated in trapezoid shape, lined with 83 black round teeth on its black apical margin (Fig. 5F). S6 lined with black border slightly curved along basal margin, apical with broadly rounded projection. S7 thin, with laterally rounded angle. S8 inverted Y-shape (Fig. 5L). Genitalia (Fig. 5N) broad. Gonoforceps bifid with dorsal medio-lateral carina pointing as acute sharp edge. Penis valve simple. Hair bright yellow to white, black hair lining on lateral area of T1–T3 and covering some basal area of T4, extending more to the median on T3 but not abutted together.

##### Floral association.

Unknown.

##### Remarks.

Since a description of male A. (R.) ignotum is given here for the first time, variations in their color pattern are discussed later in the paper (see below).

#### Anthidiellum (Ranthidiellum) phuchongensis

Taxon classificationAnimaliaHymenopteraMegachilidae

Nalinrachatakan & Warrit sp. nov.

5904B6B7-D2B5-558A-A5BE-1527D95038ED

http://zoobank.org/D7E83FBD-A9BA-4DCC-AEEA-5436A2EE699B

**Figure 3.**
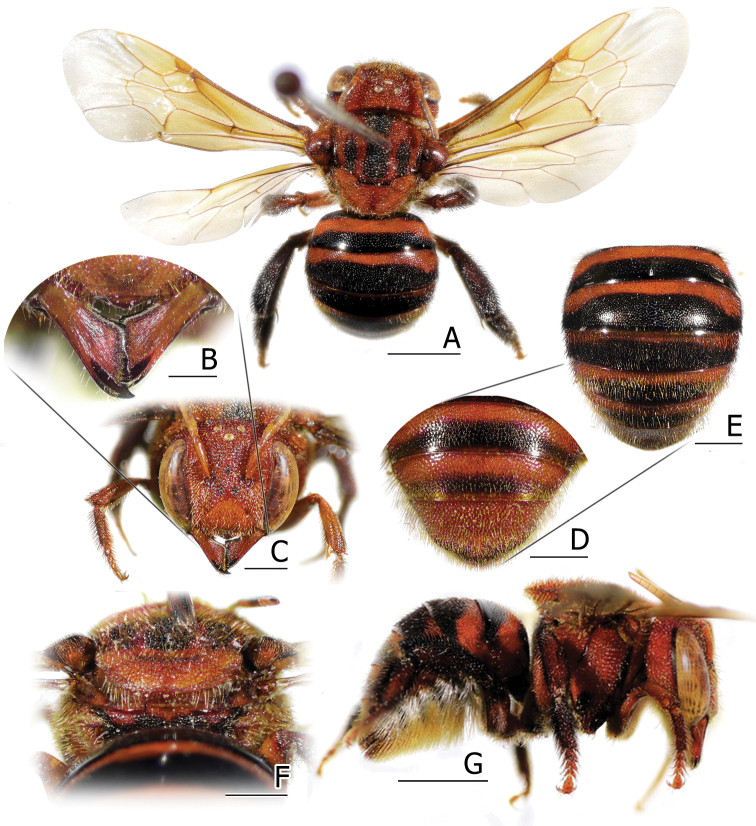
Female of *Anthidiellum phuchongensis* sp. nov. (BSRU-AA-6706) **A** dorsal habitus **B** mandible **C** face **D** metasomal apex **E** metasoma **F** propodeum **G** lateral habitus. Scale bars: 2 mm (**A, G**), 1 mm (**B, D–G**), or 0.5 mm (**C**).

**Figure 4.**
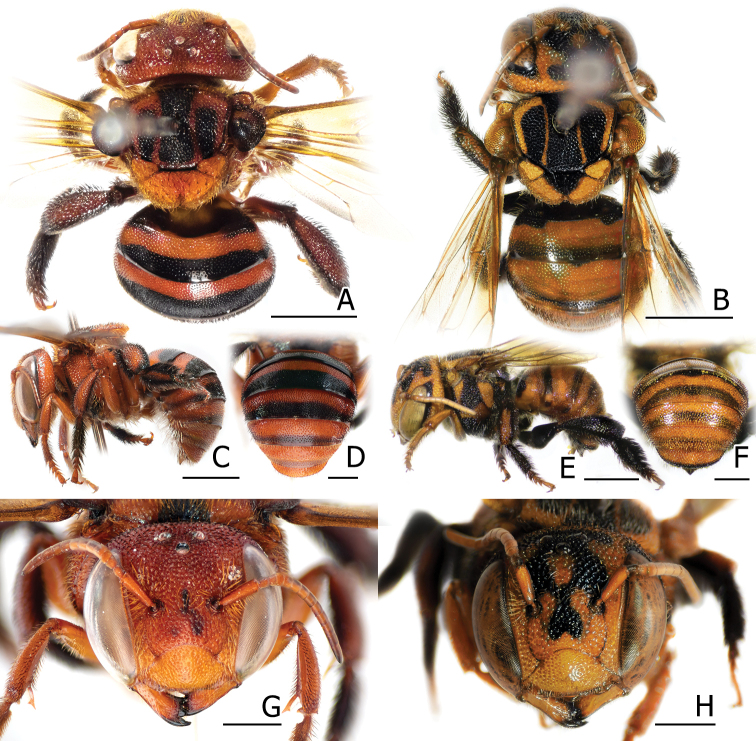
Males of *Anthidiellum phuchongensis* sp. nov. holotype (BSRU-AB-0161) (left) and *A. ignotum* Engel, 2009 (BSRU-AA-6707) (right) **A, B** dorsal habitus **C, E** lateral habitus **D, F** metasoma **G, H** face. Scale bars: 2 mm (**A, B, C, E**) or 1 mm (**D, F, G, H**).

Figs 3 , 4 (left), 5 (left)

##### Type locality.

Thailand: Ubon Ratchathani, PCNYNP, Kaeng Ka Lao [14°26'10.98"N 105°16'37.05"E, alt. 322 m]

##### Material examined

6 (5♀, 1♂). ***Holotype***: 1♂, emerged from a reared nest on 6^th^ Jan. 2020, A. Khongnak & M. Muangkam, (CUNHM: BSRU-AB-0161). ***Paratypes***: 3♀, from the same nest as holotype, emerged on 27^th^ Dec. 2019 (CUNHM: BSRU-AB-0158), 29^th^ Dec. 2019 (CUNHM: BSRU-AB-0159), and 30^th^ Dec. 2019 (CUNHM: BSRU-AB-0160).

##### Other materials.

2♀, collected on 5^th^ Jan. 2019 (CUNHM: BSRU-AA-6706) and 9^th^ Feb. 2019 (CUNHM: BSRU-AA-6936), aerial net, P. Traiyasut et al.

##### Diagnosis.

This new species resembles *Anthidiellum ignotum* Engel, 2009 in overall appearance, but differs by its dark orangish to reddish integument; facial marks restricted on the frons; black apical bands on all terga except T6, making T6 clearly orangish (Fig. 3D), whereas all other females of *Ranthidiellum* species come with black T6; black hairs on T2, T3, and lateral of T1 and T4; black hind coxa on the upper part with a small black patch around its lower part. Midleg and hindleg covered with black hairs on tibia and basal part of tarsi, making these legs superficially brownish; male S4 gradulus complete.

##### Description.

**Male holotype**: Body length 8.1 mm, head width 3.3 mm, intertegular distance 2.3 mm, wingspan 19.7 mm.

***Head*.** Orange to red-brown becoming brighter on clypeus and lower part of paraocular area; overall, sparsely covered with copperish-golden hair except black on preoccipital area and around ocelli triangle. Mandible orange, apically black. Maculation pattern showed as darker area, very obscured, similar to A. (R.) ignotum: mark on supraclypeal area [expressed as three marginal black dots, obscurely expressed in one dot while more extended for the rest (Fig. 4G)], mark along dorsal and posterior orbit, and noticeable large ovoid mark above antennal socket and stripe on ocellar triangle. Clypeus convex and depressed at apex. Clypeal punctures on lateral area coarse, becoming fine, dense, and irregular at median. Mandible apically black. Labrum yellow-orange, with conspicuous large hairs on apical surface. Scape orange to brownish. Pedicels brown. Antennal flagellum brown on 1^st^ segment, orange on 2^nd^ and 3^rd^ segment; others pale, light brown with pits on front or shiny yellow-orange surface without pits on back.

**Figure 5.**
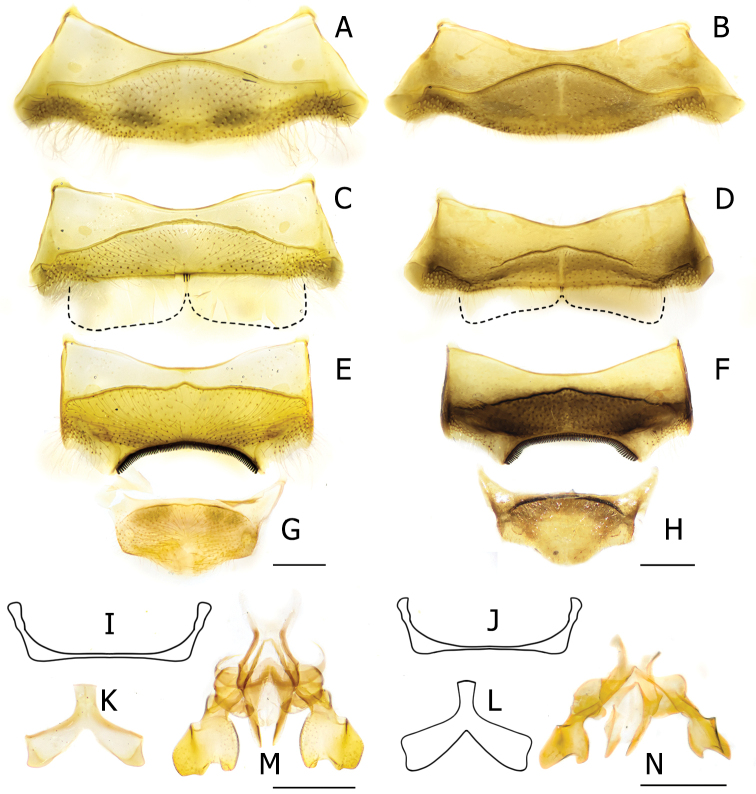
Genitalia and terminalia of male *Anthidiellum phuchongensis* sp. nov. holotype (BSRU-AB-0161) (left) and *A. ignotum* Engel, 2009 (BSRU-AA-6707) (right) **A, B** S3 **C, D** S4 **E, F** S5 **G, H** S6 **I, J** S7 **K, L** S8 **M, N** genitalia in dorsal habitus. Scale bars: 0.5 mm.

***Mesosoma*.** Covered with golden hairs. Pronotum orange, with black median stripe on anterior surface. Pronotal lobe orange, strongly carinate to lamellate. Scutum red-orange, with large black longitudinal median stripe, with two paramedial black stripes not reaching anterior and lateral margins. Scutellum yellow-orange, large, margin with median notch, median with orange area. Axilla yellow-orange. Tegula dark-brownish with anterior orange mark, somewhat translucent, with dark brown to black median mark on margin, dorsolaterally connected to inner circular mark. Metanotum orange. Propodeum extensively black except lateral orange area around propodeal spiracle. Mesepisternum anterior surface ventrally black, with orange area around lower part of inner margin. Lateral surface orange with dorsal black spots. Metepisternum without black mark except ventral stripe between midleg and hindleg.

***Wings*.** Forewing subhyaline, basally infuscated. Second recurrent vein distally joining to 2^nd^ submarginal crossvein.

***Legs*.** Overall brown-orange, darker on inner surfaces of all tibia and tarsi of midlegs and hindlegs. Foreleg somewhat darker at basitarsus and nearby tarsi. Anterior surfaces of femur and tibia of foreleg yellow-orange, exposing shiny glabrous area. Black part infused on upper part of hind coxa and small lower mark. Hair copperish-gold with black intermix on foreleg, black on midlegs and hindlegs, becoming lighter on apical tarsi, copperish-white fringe on the ventral surfaces of coxa and trochanter. Claw yellow-orange, black on both apical and subapical teeth.

***Metasoma*.** Orange with black apical band on T1–T5. T4–T5 black stripes dimmed. T6 rounded, overall orangish and lighter at apical border. T7 small, barely exposed, with acute median tooth. Hair bright gold except black on T2, T3 and lateral of T1 and T4. Sternites overall orangish, with white, dense, plumose pubescens laterally on S3–S5. S1 orangish with two lateral dark brown patches, median carina black. S2 with dark brown patches separated at median. S3 with a pair of minute dark brown patches. S4 gradulus complete, margin truncate, produced as thin transparent lobe, medially emarginated, middle of margin with three small black teeth. S5 margin black, with wide U-shaped emargination lined with a black comb of 92 blunt teeth. S6 margin produced as widely rounded lobe. S7 very narrow, with small rounded lateral lobe. S8 inverted Y-shape, basal margin strongly truncate. Apical lamina of gonoforceps enlarged, bilobed, outer lobe with prominent inner angular connected to dorsolateral carina (in A. (R.) ignotum, this angle is absent).

**Female paratype** (as in male except noted): Body length 8.2–9 (± 0.31) mm, head width 3.3–3.55 (± 0.05) mm, intertegular distance 2.5–2.8 (± 0.11) mm, wingspan 18.5–19.9 (± 0.58) mm.

***Head*.** Overall, sparsely covered with copperish-gold hair. Maculation more distinct than in male, mark on supraclypeal area expressed as three marginal black dots in paratypes (Fig. 3C), fully black inverted heart-shape mark in BSRU-AA-6936. Mandible orange and slightly reddish at apex, extensive black margin on outer ridge. Apex conspicuously broader than base, teeth black, tridentate, with large acute tooth at apex.

***Mesosoma*.** Covered with sparse copperish-gold hairs. Tegula brown-orange, with darker area at mesad. Mesepisternum black mark extended to dorsal half of anterior surfaces. Metepisternum orange with some black on anterior and posterodorsal margin.

***Legs*.** Foreleg orangish, hair copperish-gold, becoming dark brown to black apically. Midleg and hindleg orange on coxa, femur, and trochanter. The rest of midleg, except apical of tarsi, darker to brown. Hindleg dark red-brown on apical of femur, tibia, and basitarsus. Hairs copperish-gold on coxa, trochanter and apical of tarsi, dark brown to black on the rest.

***Metasoma*.** Orange with distinct black apical band on all terga, except T6. T6 obtuse. Sternite dark brown to black. Scopa yellow-gold, pale white laterally.

##### Etymology.

The name is given to the PCNYNP, Ubon Ratchathani Province, where both the holotype and paratype were collected.

##### Floral association.

Dipterocarpaceae. It is evident that *A. phuchongensis* utilized resins of *Dipterocarpus obtusifolius* Teijsm. ex Miq., a dominant plant in the area.

##### Bee kleptoparasites.

*Stelis flavofuscinular* sp. nov. (see below).

##### Remarks.

One A. (R.) phuchongensis female (BSRU-AA-6936) differs from the other paratypes in the black maculation, especially on the frons, which appeared as an inverted heart-shape, and the overall coloration was superficially darker than the other paratypes. These black extension markings are somewhat similar in female A. (R.) ignotum (see Fig. 2).

### Genus *Stelis* Panzer, 1806

#### Subgenus Malanthidium

Taxon classificationAnimaliaHymenopteraMegachilidae

Pasteels, 1969

7E310056-B8FE-5E6B-91F5-C77C89A32622

Malanthidium Pasteels, 1969: 26. Type species: Anthidium malaccense Friese, 1914, by original designation.

##### Remarks.

*Malanthidium* has an elongated body form, resembling most *Euaspis* species, and is of moderate size (8–11 mm). Only males are known. Straight subantennal suture; mandible tridentate; preoccipital margin rounded; omaulus carinated but not reaching ventral rim; distinct postero-lateral hook on axilla; scutellum large, rounded, and protruding posteriorly to overhang propodeum; wing dark brown to black; 2^nd^ recurrent vein enters distal to 2^nd^ submarginal crossvein; T6 margin with conspicuous border; S1 premarginal carina strong; S7 ventral surface smooth, margin with small median tooth.

#### Stelis (Malanthidium) flavofuscinular

Taxon classificationAnimaliaHymenopteraMegachilidae

Nalinrachatakan & Warrit sp. nov.

02FA6327-8552-5441-8165-BE040E51C92C

http://zoobank.org/AA054F28-B55D-4228-AF1A-652E032F763F

Figs 6 , 7

##### Type locality.

Thailand: Ubon Ratchathani, PCNYNP, Kaeng Ka Lao [14°26'10.98"N, 105°16'37.05"E, alt. 322 m]

##### Material examined

**2 (2**♂) **. *Holotype***: 1♂, emerged from a reared nest on the 25^th^ Dec. 2019, A. Khongnak & M. Muangkam coll. (CUNHM: BSRU-AB-0157). ***Paratype***: 1♂, same as in holotype, emerged on 23^rd^ Dec. 2019 (CUNHM: BSRU-AB-0156).

**Figure 6.**
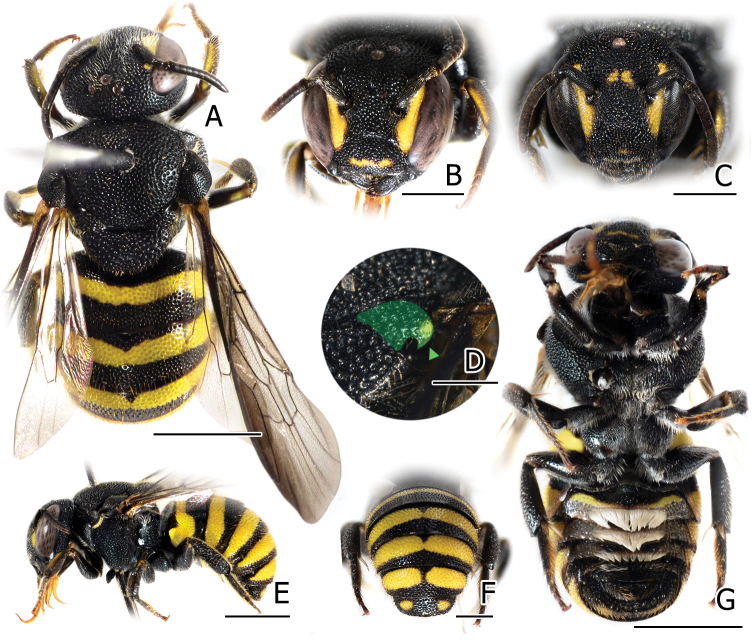
Male *Stelis flavofuscinular* sp. nov. [**A, B** holotype (BSRU-AB-0157) **C–G** paratype (BSRU-AB-0156)] showing the **A** dorsal habitus **B, C** face **D** axilla, with postero-lateral hook highlighted in green **E** lateral habitus **F** metasomal apex **G** ventral habitus. Scale bars: 2 mm (**A, E, G**), 1 mm (**B, C, F**), or 0.5 mm (**D**).

##### Diagnosis.

**Figure 7.**
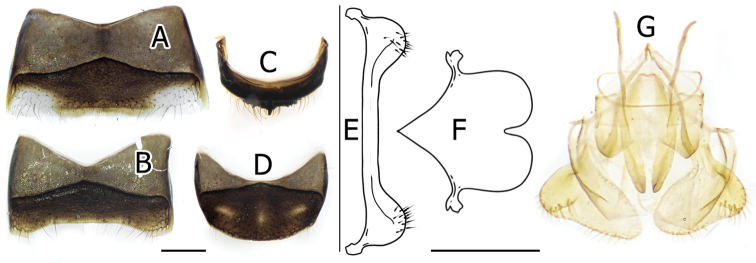
Genitalia and terminalia of male *Stelis flavofuscinular* sp. nov. (BSRU-AB-0157) **A** S4 **B** S5 **C** T7 **D** S6 **E** S7 **F** S8 **G** genitalia coupled with S8 in ventral habitus. Scale bars: 0.5 mm.

With only males known, *Stelis flavofuscinular* is distinct from its only known congener, *S. malaccensis* from Malaysia, as follows: head overall black, with yellow paraocular mark reaching close to the top of eyes, and narrow mark restricted close to apical area of clypeus; antennal scape black; Mesosoma overall black except yellow on postero-lateral hook of axilla; T1–T5 with large yellow strike band, with little median disruption that is pronounced more on rear metasomal segments; T6 with lateral yellow dots; S2–S4 with distinct median patch of long white hairs, while lacking black midapical comb. S4 and genitalia as in Fig. 7G.

##### Description.

**Male holotype**: Body length 8.2 mm, head width 2.4 mm, intertegular distance 2.1 mm, wingspan 16.1 mm. **Paratype**: Body length 8.1 mm, head width 2.5 mm, intertegular distance 2.0 mm, wingspan 15.9 mm.

***Head*.** Largely black, overall covered with sparse pale white hairs. Mandible black with red-brown infused, tridentate, with apically large acute tooth. Labrum black with rounded margin. Clypeus black with narrow yellow stigma (obscured in BSRU-AB-0156) on surface close to apex, punctures very dense, separated with less than half of its diameter, margin truncate, with small inconspicuous median tubercle. Subantennal suture strait. Frons punctures coarser than on clypeus, with two yellow stigmas (Fig. 6B), typically very obscured but can be recognized in BSRU-AB-0157, present above area between antennal socket. Paraocular area with yellow mark extending along orbit close to top of ommatidia. Interocellar distance shorter than ocellooccipital distance. Vertex and preoccipital area with coarse and dense punctures, with distinct microsculptures. Surfaces nearby lateral ocelli shiny glabrous, with fewer punctures, whereas fine and dense around middle ocelli. Scape and pedicels brown-black, frontal surface covered with dense pale white hairs. Antennal flagellum dark brown, F1 color lighter in apical half, F2 almost broader than long.

***Mesosoma*.** Overall black, covered with sparse pale white hairs. Pronotal lobe rounded. Omaulus carinated but does not reach venter of thorax. Mesepisternum swollen laterally, with fine dense punctures on anterior surface, very coarse and dense on lateral surface.

Scutum with coarse, dense punctures, separated by about half of its diameter, becoming fine and dense posteriorly. Scutellum rounded, extended posteriorly to overhang propodeum, punctation rather sparse in comparison with scutum. Scutoscutellar suture open, divided into two shiny bottom foveae. Axilla black (Fig. 6D), postero-lateral hook yellow, punctures fine and dense. Scutum, scutellum, and axilla come with distinct microsculptures. Tegula large, dark brown to black, with very fine, dense punctures. Propodeum black, median area shiny glabrous, with distinct fovea behind spiracle.

***Wings*.** Dark brown to black especially on anterior half of forewing, and marginal cell. Stigma black. Second recurrent vein enters distal to 2^nd^ submarginal crossvein, separating medial vein in 4:1 ratio.

***Legs*.** Overall black-brown, with restricted yellow maculation present on anterior surface of tibia and apical femur of foreleg, dorsal surface of apical femur and basal tibia of midleg. Fore and mid tibia apically with two outer apical spines. Hind tibia apically with outer rounded projection. Tibial spur pale, bifid on foreleg. Hairs pale white, brown on tarsi. Hind basitarsus black, inner surfaces with brown dense hair fringe. Claw red-brown, apically black on hind tarsi. Arolia present, light brown.

***Metasoma*.** Overall black with yellow maculation. Tergites covered with sparse, short, brownish hair, punctation coarse, separated by its diameter, uniformly distributed but somewhat confused on T6. T1–T5 with large yellow strike bands, with little median disruption that is pronounced more in rear metasomal segments. T6 large with lateral dots, apical margin rounded, carinated, forming ventral border. T7 small, marginal area depressed, median area of apical margin broadly crenulate with distinct median erected tooth that making lateral shallow emargination, ventral surface smooth with lateral angle making T7 weakly tridentate. Sternite black, with scattered brownish hairs. S1 median carina strong, premarginal carina strong, extended ventrally but not clearly overhanging margin. S2–S4 (Fig. 6G) laterally translucent, with distinct median white pubescent erected from premarginal band. S2 and S3 with yellow premarginal bands, but very narrow and medially restricted in the latter. S5 widely emarginated, with very sparse white pubescent. S6 margin rounded. S7 very narrow, ventral surface smooth, lateral margin with extended rounded lobe with dispersed erected hairs. S8 (Fig. 7F) very clear apically, extending to two rounded apical lobes separated with median U-shape notch, resembling inverted heart shape. Genitalia as in Fig. 7G.

##### Etymology.

The word *flavo* means “yellow”, while *fuscinular* means “hook”. Thus, the specific epithet, *flavofuscinular*, principally refers to the yellow postero-lateral axilla hook of male bees that contrasts with its overall black mesosoma.

##### Bee host.

*Anthidiellum phuchongensis* sp. nov. (see above). It is possible that *S. flavofuscinular* sp. nov. may also be a cleptoparasite of other *Megachile* species that are also frequently encountered in the PCNYNP area. Kasparek (2015) suggested that the hosts of *Stelis* species are mainly members of Megachilinae, and some *Stelis* species have a wide range of hosts.

##### Floral association.

Unknown.

##### Remarks.

Though the color pattern observed on the mesosoma and metasoma seems invariant, there are some variations in the yellow maculation especially on the face, noticeable in two specimens possibly from the same cohort, and so it is likely to have a greater level of variation in the population. Stelis (M.) malaccensis (Friese, 1914), redescribed by Pasteels (1969), differs mainly in coloration. It exhibits very dense punctures over all the thorax, a more subtriangular scutellum, yellow markings on the base of the mandible, scape, supraclypeal area, paraocular area, preoccipital area, vertex, mesopleuron, anterolateral margin of the scutum, and margin of the scutellum and axilla.

#### Key to female Anthidiellum (Ranthidiellum) species of the world

Modified from Engel (2009), see discussions below.

**Table:** 

1	Face mostly without black area, if present, restricted to frons; metasoma largely reddish, orangish, or ferruginous	**2**
–	Face with extensive black areas; metasoma largely black, dark brown, or dark ferruginous	**3**
2	T6 black; body ferruginous; T1–T5 without distinct black apical band, sometimes with black stigma infused (Fig. 2)	**A. (R.) ignotum Engel, 2009**
–	Body including T6 orangish (Fig. 3D); T1–T5 with prominent black apical band (Fig. 3)	**A. (R.) phuchongensis sp. nov.**
3	Mesoscutum overall black; head black with clypeus, mandible, and antenna orangish to reddish; T6 covered with white to yellowish plumose tomentum; forewing conspicuously dark brown at basal half, apically hyaline	**4**
–	Mesoscutum with reddish to orangish anterolateral L-shape mark; head with more extensive lighter orangish to reddish area, especially on paraocular area along the inner and outer orbits, and oval mark below middle ocelli; T6 not covered with plumose tomentum; forewing without conspicuous dark-brown area	**A. (R.) meliponiforme (Cockerell, 1919)**
4	Metasoma black, with red-brown to black infused basally on T1–T5; scutellum and axilla with narrow orangish to reddish marginal band	**A. (R.) rufomaculatum (Cameron, 1902)**
–	Metasoma dark brown to black, with orangish to reddish band present apically on T1–T5; band on the scutellum, and axilla margin broader	**A. (R.) apicepilosum (Dover, 1929)**

#### Key to male Anthidiellum (Ranthidiellum) species of the world

The characters of male A. (R.) rufomaculatum (Cameron, 1902) and A. (R.) apicepilosum (Dover, 1929) are based on Pagden (1934) and Pasteels (1969). Male A. (R.) meliponiforme (Cockerell, 1919) remains unknown.

**Table:** 

1	Metasoma largely reddish, orangish, or ferruginous	**2**
–	Metasoma largely black, dark brown, or dark ferruginous	**3**
2	Body integument ferruginous (Fig. 4 right); face with extensive black area (Fig. 4H); Tergal apex translucent, covering black basal band of its successor; S4 gradulus incomplete (Fig. 5D)	**A. (R.) ignotum Engel, 2009**
–	Body integument orangish to reddish (Fig. 4 left); face with small black marks restricted on the frons (Fig. 4G); Tergal apex almost opaque, T1–T5 with black marginal band; S4 gradulus complete (Fig. 5C)	**A. (R.) phuchongensis sp. nov.**
3	Metasoma uniformly dark red-brown, dark brown, or black, sometimes with broad reddish apical margins; T6 covered with plumose white tomentum; S5 apical comb with “± 80 teeth”	**A. (R.) rufomaculatum (Cameron, 1902)**
–	Metasoma dark brown to black, with metallic reddish reflections infused apically predominantly on second and third segments; T6 not covered with white tomentum; S5 apical comb with “over 60 teeth”	**A. (R.) apicepilosum (Dover, 1929)**

## Discussion

### Taxonomic implications

It appears that sexual dimorphism in coloration is very strong in A. (R.) ignotum but very weak in A. (R.) phuchongensis. Both species are very similar in their morphology, but differ in their sternal and genitalic structures. The dorsolateral carina of the gonoforceps is present in both species, but it is still unclear whether this character is present in other *Ranthidiellum* species as the character is never reported. The genitalia descriptions and illustrations of A. (R.) apicepilosum (Dover, 1929) and A. (R.) rufomaculatum (Cameron, 1902) are vague (see Pagden 1934; Pasteels 1972), and males of A. (R.) meliponiforme (Cockerell, 1919) are unknown, though Ascher et al. (2016, see fig. 5A) reported an unknown male specimen of *Ranthidiellum* from eastern Cambodia that is presumed to be A. (R.) meliponiforme.

Color variations in *Ranthidiellum* are poorly understood as they are rarely found (Soh et al. 2016). In our study, although only six A. (R.) ignotum females were examined, several color variations were detected. We arbitrarily categorized these specimens into two forms based on the variations in the infused black integument as “normal” and “less melanized” forms (Fig. 2). One female collected from Phayao Province (BSRU-AA-1250) had “less melanized” traits, where the midleg, hindleg, and the anterior part of the scutum had reduced black areas. In addition, as the expression of the black pattern declined, the lateral black stripes on the anterior parts of the scutum appeared as red-brown. The “normal” form specimens had varying extensions of black areas, some obviously extended to the anterior surfaces of the mesonotum, propodeum, and anterior surfaces of T1. Also, more infused black marks were prominent on the face and T1–T5. One specimen from Chiang Mai (BSRU-AA-6709) showed a black triangular mark on the scutellum (Fig. 2D). It is noteworthy that this type of color variation can also be detected in A. (R.) phuchongensis.

We also examined the photographs of *Ranthidiellum* holotypes deposited at NHMUK: A. (R.) apicepilosum Dover, 1929, A. (R.) meliponiforme (Cockerell, 1919), and A. (R.) rufomaculatum (Cameron, 1902). All types had labels showing “TYPE (POSSIBLE)”, and the labels were in accord with the original descriptions. Despite the type of A. (R.) rufomaculatum being labeled “Selected as types, Pasteels”, the redescription by the author (Pasteels 1969) was not congruent with the material itself in some aspects. For example, Pasteels (1969, p 124, “Couleur” section) noted “En rogue ferrugineux … de larges bandes sur les tergites 1–5 (les deux dernières jaunâtres)” [reddish-ferruginous band on T1–T5, with the last two yellowish], while Cameron’s (1902) original description and Mavromoustakis’s (1936) notes are vague and did not mention any terga band. The character was shown in the material as obscured red-brown to black area infused basally for all denoted terga, thus, yellowish color stated by Pasteels should be a vague interpretation led by the distinct yellow tomentum, while color information can lead to misidentification since this will easily fit with the reddish-ferruginous broad apical band founded in A. (R.) apicepilosum. Materials of A. (R.) apicepilosum and A. (R.) rufomaculatum appear very similar in appearance, especially facial and mesosoma maculation (see Table 1), though these comparisons are based on very limited material. Considering current evidence presented with the synopsis of *Ranthidiellum* species (see Table 1), we revised and updated the identification keys based on Engel (2009) to both male and female species.

**Table 1.** List of Anthidiellum (Ranthidiellum) and Stelis (Malanthidium) of the world. (***e***: emergence record; ***f***: flight record; ***t***: type locality).

Species	Original description	Supplementary literature	Documented localities	Phenology notes
*A. apicepilosum* (Dover, 1929)	Dover 1929	Pagden 1934; Pasteels 1969, 1972	Thailand (Nakhon Si Thammarat ***t***), Malaysia (Gunung Angsi, Negeri Sembilan; Batu Ferringhi, Penang; Bukit Kutu, Selangor)	February (24^th^***f***); March (8^th^***e***, 11***e***); April (15^th^***f***); August (24^th^***f***)
*A. ignotum* Engel, 2009	Engel 2009	Soh et al. 2016	Thailand (Chiang Mai, Nakhon Ratchasima ***t***, Phayao)	June (1^st^***f***); July (10^th^***f***); December (9^th^***f***, 13^th^***f***, 19^th^***f***: possibly mating flight)
*A. meliponiforme* (Cockerell, 1919)	Cockerell 1919	Pagden 1934; Pasteels 1969, 1972	Malaysia (Sandakan, Sabah, Borneo ***t***), Cambodia?* (Keo Seima)	not indicated
*A. phuchongensis* sp. nov.	this study	–	Thailand (Ubon Ratchathani)	January (5^th^***f***, ***e***, 6^th^***f***); February (9^th^***f***); October (10^th^, observed in habitat survey); December (27^th^***e***, 29^th^***e***, 30^th^***e***)
*A. rufomaculatum* (Cameron, 1902)	Cameron 1902	Mavromoustakis 1936; Pasteels 1969, 1972	Malaysia (Kuching, Sarawak, Borneo ***t***), Indonesia (Sumatra)	April (30^th^***f***)
*S. malaccensis* (Friese, 1914)	Friese 1914	Pasteels 1969; Michener and Griswold 1994; Michener 2007	Malaysia (Taiping Hill, Perak ***t***)	February (-)
*S. flavofuscinular* sp. nov.	this study	–	Thailand (Ubon Ratchathani)	December (emerge: 23^rd^***e***, 25^th^***e***)

*identified as *A. meliponiforme* in affinity (see Ascher et al. 2016).

Michener (2007)’s diagnosis of *Stelis* denoted that males commonly have a midapical comb on S4, which is usually used as a diagnosis character. However, in the S. (M.) flavofuscinular sp. nov. described herein the midapical comb on S4 was absent. Besides S4, the studies on the genitalia and other hidden terga are very difficult to perform, with very few studies containing illustrations of these structures as mentioned in comprehensive revisions of *Stelis* by Kasparek (2015). It is very important to carefully prepare the genitalia and associated sclerites to deliver more comprehensive and accurate data.

### Association of Stelis (M.) flavofuscinular sp. nov. with Anthidiellum (R.) phuchongensis sp. nov.

**Figure 8.**
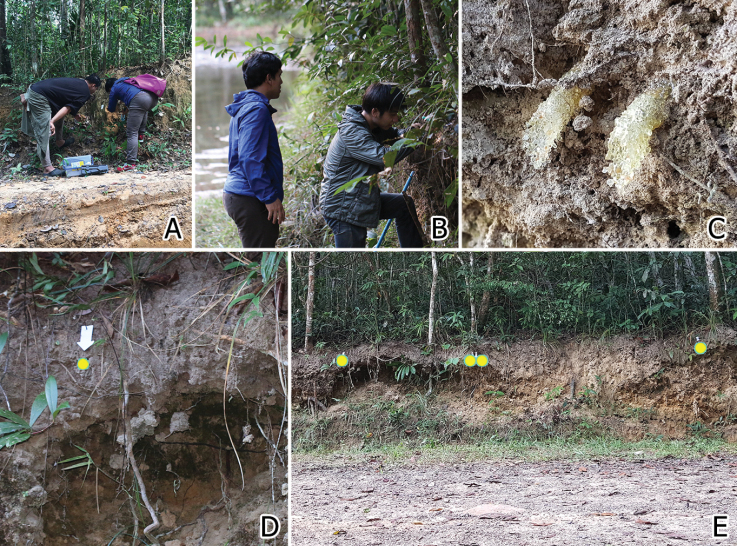
Nesting habitat of *Anthidiellum phuchongensis* sp. nov. at PCNYNP, Ubon Ratchathani Province, Thailand **A, B** nest excavation process and area **C** resinous nest entrances **D, E** nest locations of *A. phuchongensis* on vertical earth bank (highlighted with yellow dots).

At the PCNYNP, A. (R.) phuchongensis putatively constructed their nests in preexisting cavities, mostly from abandoned mygalomorph spider nests that are abundant in the sandy earth bank, making protruding translucent resinous entrance tubes that curved downwards (Fig. 8C), similar to the nest described for A. (R.) apicepilosum, which utilize deserted potter wasp nests in Malaysia (Pagden 1934; Pasteels 1972).

**Figure 9.**
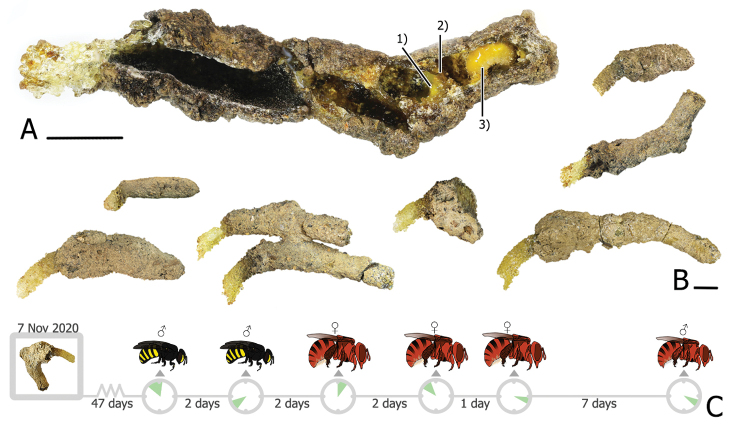
Nest structure of *Anthidiellum phuchongensis* sp. nov. **A** longitudinal dissected nest: 1) provisional mass, 2) resinous partition, and 3) predefecated larvae **B** showing diversity of nest morphology **C** timeline of bees emerging from the reared nest. Scale bars: 1 mm.

This is the first report on the host-cleptoparasite relationship in *Ranthidiellum*. The Anthidiellum (R.) phuchongensis nest collected in December 2019 was maintained under a laboratory condition for 47 d until the first adult bee, a male Stelis (M.) flavofuscinular sp. nov., emerged, followed by another male 2 d later and then three A. (R.) phuchongensis females and a male over the remaining 12 d (Fig. 9C).

*Stelis* is known to adopt at least two strategies in attacking host cells (Litman 2019): the female S. (Dolichostelis) sp. is reported to attack closed host cells and to destroy the host offspring before laying eggs (Parker et al. 1987), whereas *S.* (*Stelis* s. str.) attacks open host cells to lay eggs and let the emerged larvae then kill the host eggs or larvae (Rust and Thorp 1973; Torchio 1989; Rozen and Hall 2011). Recent phylogenetic studies (Litman et al 2013, 2016) suggested S. (Malanthidium) to be more closely related to *S.* (*Stelis* s. str.) than to S. (Dolichostelis), and assumed that S. (Malanthidium) must be an open-cell attacker. Our work found evidence to suggest that S. (M.) flavofuscinular might be an open-cell attacker, since the host nest had no indication of resin modification by the parasite.

Taxonomic knowledge on *Stelis* in Southeast Asia is very scant (Michener 2007). Historically, there is only one species described: S. (M.) malaccensis (Friese, 1914), from Taiping hills, Perak, Malaysia (originally noted as “Taiping Hill, Malakka” by von Buttel-Reepen), previously a monobasic for *Malanthidium*. In this study, S. (M.) flavofuscinular is the second described *Malanthidium* species (see Table 1). Michener and Griswold (1994) and Michener (2007) also pointed out that there are at least two additional undescribed *Malanthidium* species, but the details were not provided.

## Supplementary Material

XML Treatment for Subgenus Ranthidiellum

XML Treatment for Anthidiellum (Ranthidiellum) ignotum

XML Treatment for Anthidiellum (Ranthidiellum) phuchongensis

XML Treatment for Subgenus Malanthidium

XML Treatment for Stelis (Malanthidium) flavofuscinular
